# Durable biochemical and radiographic response during toripalimab treatment in MSI-high/TMB-high mCRPC: a case report

**DOI:** 10.3389/fonc.2026.1907524

**Published:** 2026-10-09

**Authors:** Junhang Ding, Zhengxin Liu, Jinyu Tian, Feng Wang, Chuan Liu

**Affiliations:** 1 Department of Urology, The Second Affiliated Hospital of Chongqing Medical University, Chongqing, China; 2 Department of Urology, Traditional Chinese Medicine Hospital of Fengjie, Fengjie, Chongqing, China

**Keywords:** case report, high microsatellite instability, high tumor mutational burden, immunotherapy, metastatic castration-resistant prostate cancer, PD-1 inhibitor, toripalimab

## Abstract

We present a 79-year-old male patient with MSI-H/TMB-H (91.5 mutations/Mb) metastatic castration-resistant prostate cancer who progressed following abiraterone and enzalutamide treatment. When toripalimab was started, his serum PSA was 102 ng/mL. We maintained androgen-deprivation therapy with leuprorelin and stopped enzalutamide. Toripalimab 240 mg was given intravenously every 21 days. His PSA dropped below 0.09 ng/mL after three treatment cycles. We retrospectively applied RECIST version 1.1 criteria and selected the locally invasive prostate-soft-tissue mass as our only measurable non-nodal target lesion. Its maximal diameter shrank from 119 mm at baseline to 43 mm after six cycles (63.9% reduction), and further to 31 mm after 25 cycles (73.9% reduction), satisfying criteria for partial response. Pelvic lymph nodes could only be classified as non-target disease because short-axis diameters were unavailable in imaging reports, though these nodes resolved on imaging after six cycles. Bone lesions were not included in RECIST response evaluation and were interpreted separately on serial bone scans. At cycle 31 (roughly 21 months after toripalimab commencement), PSA stayed undetectable without clinical or radiological signs of progression; the biochemical response persisted for about 19 months starting from cycle 3. As of manuscript revision, the patient remains on toripalimab plus leuprorelin with routine follow-up. No treatment-related adverse events were identified using CTCAE version 5.0. We also conducted a targeted narrative review of mCRPC cases treated with PD-1/PD-L1 inhibitors other than pembrolizumab. This single-case observation highlights the value of genomic profiling for mCRPC patients with relevant biomarkers. Readers should interpret these findings as hypothesis-generating and not proof of universal toripalimab activity.

## Introduction

Prostate cancer is the second most frequently diagnosed cancer and the fifth leading cause of cancer death among men worldwide, with approximately 1.5 million new cases and 397,000 deaths estimated in 2022 (1). In China, epidemiologic data have also documented an increasing prostate cancer burden over recent decades (2). Metastatic castration-resistant prostate cancer (mCRPC) is defined by disease progression despite castrate levels of testosterone and remains clinically challenging because of primary or acquired resistance to systemic therapy (3). Programmed death-1 (PD-1)/programmed death-ligand 1 (PD-L1) inhibitors have transformed the treatment of multiple malignancies, but activity in unselected mCRPC is limited (4). The clinical relevance of immune checkpoint blockade in prostate cancer is therefore principally biomarker-driven. In particular, MSI-H, which commonly reflects deficient DNA mismatch repair, and TMB-H can identify tumors with increased immunogenicity, and current guideline use of checkpoint inhibition in prostate cancer emphasizes molecular selection rather than mCRPC status alone (6, 7). Toripalimab is a PD-1 inhibitor widely used in several malignancies but is rarely reported in prostate cancer. A previous report described PSA normalization after toripalimab in mCRPC but focused on an immune-related adverse event and did not provide a detailed radiographic response assessment (5). Here, we describe a biomarker-selected patient with MSI-H/TMB-H mCRPC who achieved durable biochemical and radiographic disease control during toripalimab treatment, together with a targeted review of published non-pembrolizumab PD-1/PD-L1 inhibitor reports.

## Case presentation

A 79-year-old male initially presented with dysuria and was admitted in September 2022. He had a history of type 2 diabetes mellitus and emphysema, with well-maintained glycemic control over a prolonged period. Upon admission, his PSA level was 22.50 ng/mL, and serum testosterone was 762.00 ng/dL. Digital rectal examination revealed a hard, fixed prostate with an irregular contour and loss of the median sulcus. Prostate magnetic resonance imaging (MRI) indicated possible bilateral seminal vesicle invasion and enlarged lymph nodes in the right pelvic sidewall (**Figure 1A**). A transrectal ultrasound-guided prostate biopsy confirmed prostatic adenocarcinoma with a Gleason score of 5 + 5 = 10. Whole-body bone scintigraphy showed no evidence of metastasis (**Figure 2A**). The clinical staging was determined as T3bN1M0 (Stage IVa). The patient declined surgical intervention. Initial treatment included leuprorelin acetate sustained-release microspheres (3.75 mg subcutaneously monthly), abiraterone acetate (1000 mg orally daily), and prednisone (5 mg orally twice daily).

**Figure 1:**
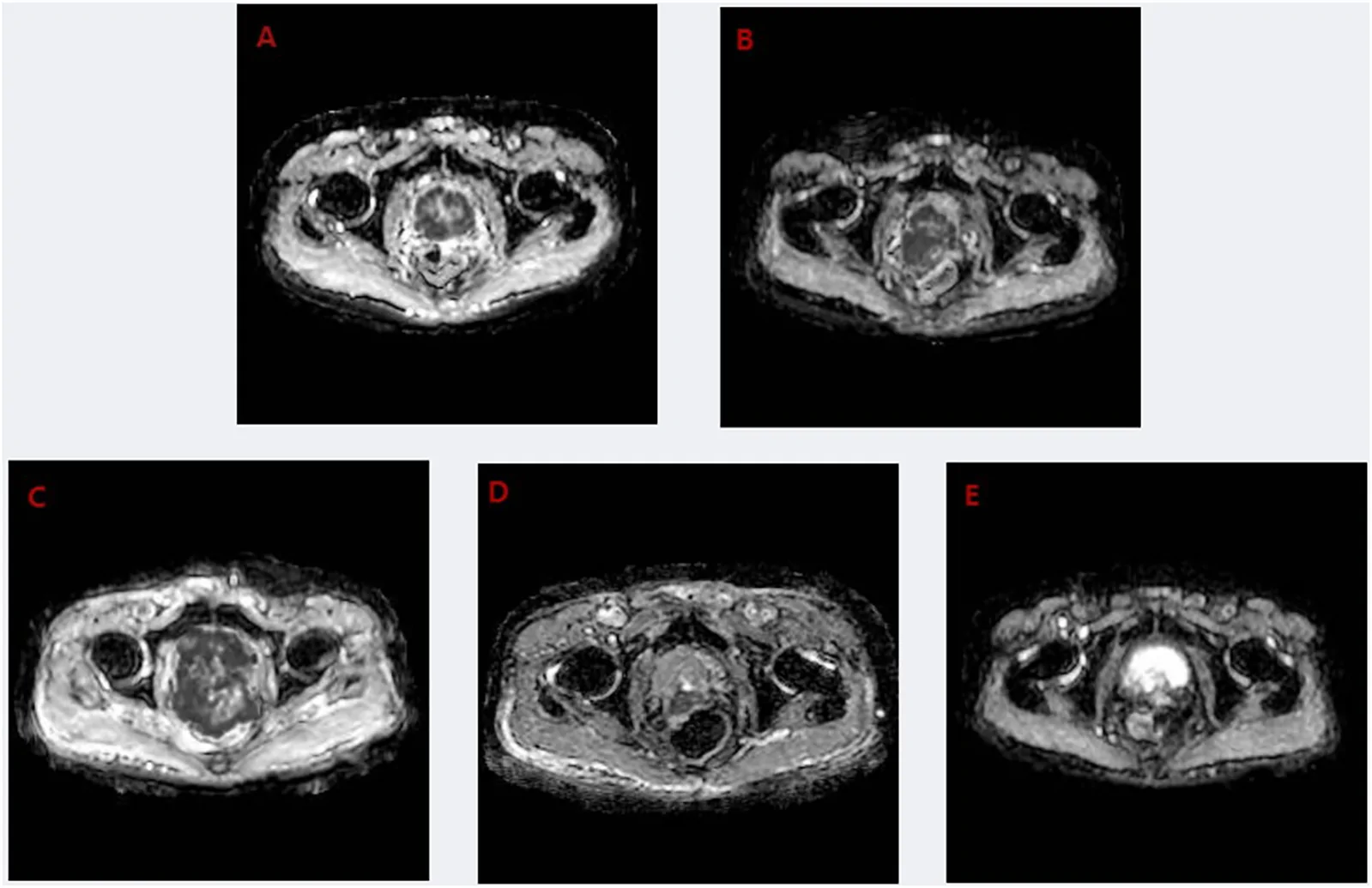
Representative prostate MRI at different disease stages. **(A)** September 2022. Baseline (initial admission). Prostate: 63 × 59 × 81 mm. **(B)** July 2023. First progression. Prostate: 45 × 71 × 64 mm. **(C)** November 2023. Pre-toripalimab RECIST baseline. Prostate/locally invasive soft-tissue lesion: 109 × 95 × 119 mm (maximum reported diameter 119 mm). **(D)** April 2024. MRI after six documented toripalimab cycles. Prostate/locally invasive soft-tissue lesion: 43 × 23 × 31 mm. **(E)** June 2025. MRI after 25 toripalimab cycles. Prostate/locally invasive soft-tissue lesion: 31 × 20 × 22 mm.

**Figure 2:**
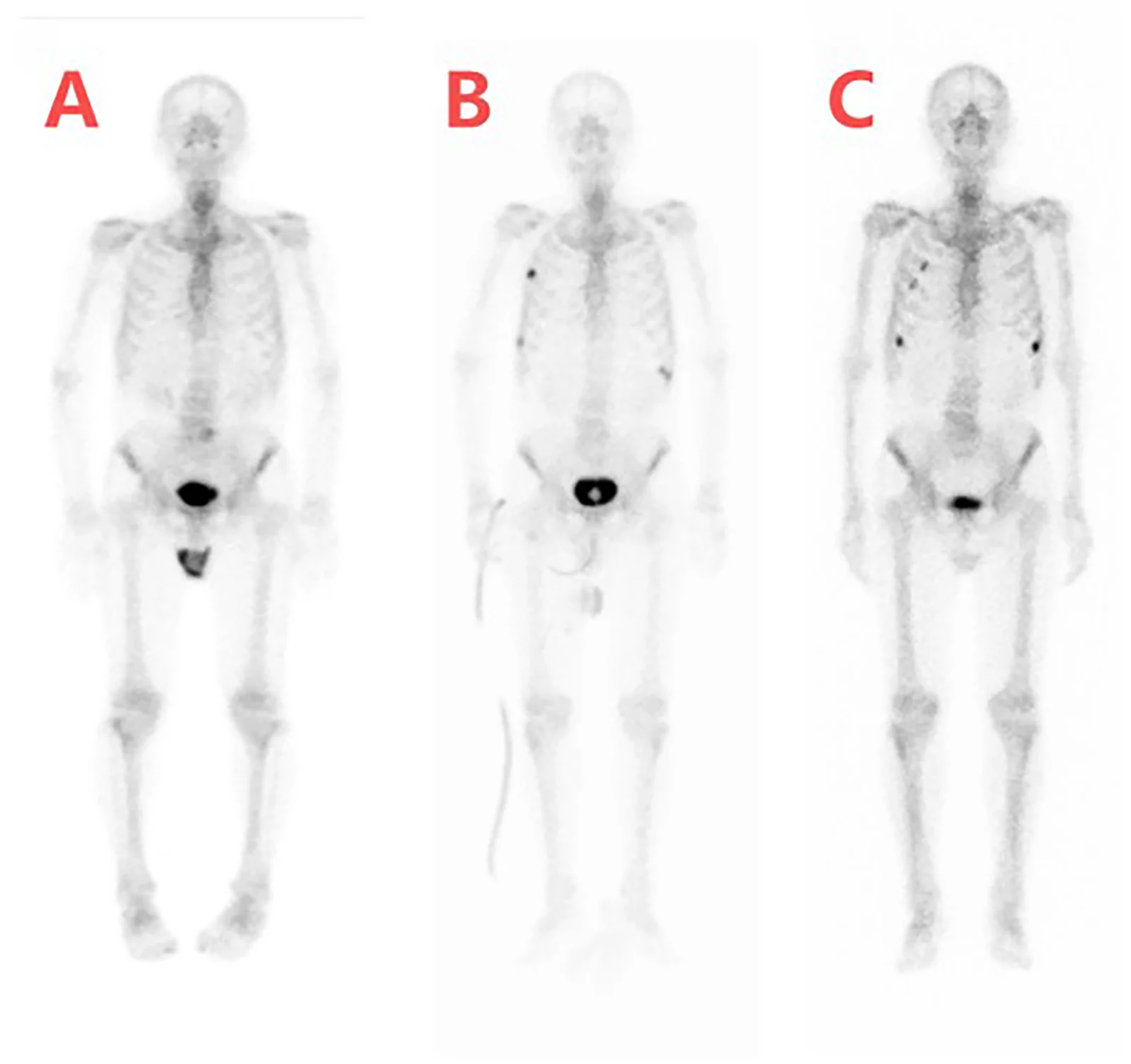
Serial whole-body bone scans.​ **(A)** September 2022. Baseline (initial admission): 1.No definite osseous metastasis. 2.L5 degenerative changes. **(B)** July 2023. First progression (before immunotherapy): 1. New foci: right 4th/8th ribs and left 8th-9th anterior ribs, suspicious for metastases on bone scintigraphy; no contemporaneous CT, MRI, SPECT/CT, or PSMA PET/CT confirmation was available. 2. Stable L5 degeneration. **(C)** June 2025. Post-25 immunotherapy cycles (approximately 18 months): 1. Newly visualized foci in the right 3rd/7th anterior ribs and left 7th anterior rib, interpreted as being consistent with old fractures rather than new metastatic lesions. 2. Right 4th and 8th anterior rib foci: increased density with reduced radiotracer uptake. 3. Previously suspicious right 8th and left 8th-9th anterior rib foci: normalization of radiotracer uptake.

In July 2023, the patient developed gross hematuria of unknown etiology, accompanied by urinary frequency, urgency, and obstructive voiding symptoms requiring urinary catheterization. Repeat PSA testing showed a level of 20.90 ng/mL, with serum testosterone <5 ng/dL. Whole-body bone scintigraphy revealed three new foci in the right 4th and 8th ribs and the left 8th-9th anterior ribs that were suspicious for metastases (**Figure 2B**). These rib foci were not independently confirmed at that time by CT, MRI, SPECT/CT, or PSMA PET/CT. We therefore describe them as suspicious bone-scan findings rather than definitive RECIST-evaluable metastases. Prostate MRI demonstrated increased lesion size with internal hemorrhage, more extensive seminal vesicle invasion, possible invasion of the bladder posterior wall and rectal anterior wall, and markedly enlarged pelvic lymph nodes (**Figure 1B**). In the setting of radiographic and symptomatic progression despite castrate testosterone, the disease was considered castration-resistant, with suspected osseous metastases. Abiraterone acetate with prednisone was discontinued, and enzalutamide 160 mg orally once daily was initiated while monthly leuprorelin 3.75 mg was continued. Because severe hematuria and obstructive voiding symptoms substantially affected quality of life, the patient underwent palliative transurethral resection of the prostate (pTURP), with approximately 80 g of prostatic tissue resected. Resected prostate tissue and peripheral blood samples were collected for comprehensive genomic profiling. The assay report identified genomic alterations in AR, PIK3CA, BRIP1, RAD50, ATM, FBXW7, and CDK12, together with TMB-H (91.5 mutations/Mb) and MSI-H.

In November 2023, the patient experienced recurrent obstructive voiding symptoms complicated by acute urinary retention. Urinalysis confirmed a severe complicated urinary tract infection, occurring concurrently with objective disease progression. Enzalutamide had been administered at 160 mg/day from July to November 2023 (approximately four months). The patient reported taking enzalutamide every day as prescribed and denied missed doses; no dose reduction or treatment interruption was documented. During this interval, PSA increased from 20.90 to 102 ng/mL, and dynamic contrast-enhanced prostate MRI showed a substantial increase in the prostate lesion, more extensive invasion of the seminal vesicles, bladder wall, and anterior rectal wall, enlarged pelvic metastatic lymph nodes, and new bilateral iliac bone metastases (**Figure 1C**), providing biochemical and radiographic evidence of progression while receiving enzalutamide. Given the suboptimal outcome following pTURP, a percutaneous suprapubic cystostomy was performed after adequate treatment of the urinary tract infection to relieve refractory voiding symptoms. Based on genomic profiling indicating MSI-H and TMB-H, immune checkpoint inhibitor therapy was subsequently considered. Following discussion of available options, the patient and his family chose intravenous toripalimab (Tuoyi; Junshi Bio, Shanghai, China) at 240 mg every 21 days. Leuprorelin 3.75 mg subcutaneously every 4 weeks was continued to maintain androgen deprivation, whereas enzalutamide was discontinued at toripalimab initiation. The patient had an Eastern Cooperative Oncology Group Performance Status (ECOG PS) score of 1 before starting toripalimab.

After three cycles of toripalimab, PSA decreased below the detection limit (<0.09 ng/mL), while testosterone remained <5 ng/dL. After six cycles, dysuria and hematuria had improved substantially. Repeat MRI showed that the reported dimensions of the locally invasive prostate/soft-tissue lesion decreased from 109 × 95 × 119 mm before toripalimab to 43 × 23 × 31 mm; the maximum reported target-lesion diameter therefore decreased from 119 to 43 mm (63.9%). The extent of seminal-vesicle, bladder posterior-wall, and rectal anterior-wall invasion was markedly reduced, and the previously enlarged pelvic lymph nodes were no longer radiographically evident (**Figure 1D**). Bilateral iliac bone metastases remained radiologically stable. The suprapubic catheter was subsequently removed, and unobstructed spontaneous voiding was restored.

After 25 treatment cycles, MRI showed further reduction of the locally invasive prostate/soft-tissue lesion to 31 × 20 × 22 mm, corresponding to a decrease in maximum reported target-lesion diameter from 119 mm at the pre-toripalimab baseline to 31 mm (73.9%). Pelvic nodal non-target disease remained resolved, and bilateral iliac bone metastases remained radiologically stable (**Figure 1E**). With a decrease of at least 30% in the single designated target-lesion diameter and no unequivocal progression of non-target soft-tissue disease, the RECIST version 1.1 soft-tissue response was classified as a partial response. Bone lesions were not used to determine RECIST response and were evaluated separately. Follow-up whole-body bone scintigraphy showed reduced or normalized radiotracer uptake in the previously suspicious rib foci, with no new metastatic bone lesions identified; newly visualized rib foci were interpreted as being consistent with old fractures (**Figure 2C**). The patient also had sustained improvement in obstructive voiding symptoms and hematuria.

Following 31 treatment cycles (approximately 21 months after toripalimab initiation), the patient’s PSA level remained undetectable (<0.09 ng/mL), with no clinical symptoms or radiographic evidence of disease progression (**Figure 3**). Because the biochemical response was first documented after cycle 3 and remained present through cycle 31, its documented duration was approximately 19 months. Throughout the reported treatment course, toripalimab was administered regularly at 240 mg every 21 days, with no dose modification or treatment interruption. At the time of manuscript revision, the patient continues toripalimab every 3 weeks and monthly leuprorelin, returns regularly for follow-up, and will remain under longitudinal surveillance. No treatment-related adverse events were recorded during serial clinical assessments according to the Common Terminology Criteria for Adverse Events (CTCAE) version 5.0.

**Figure 3:**
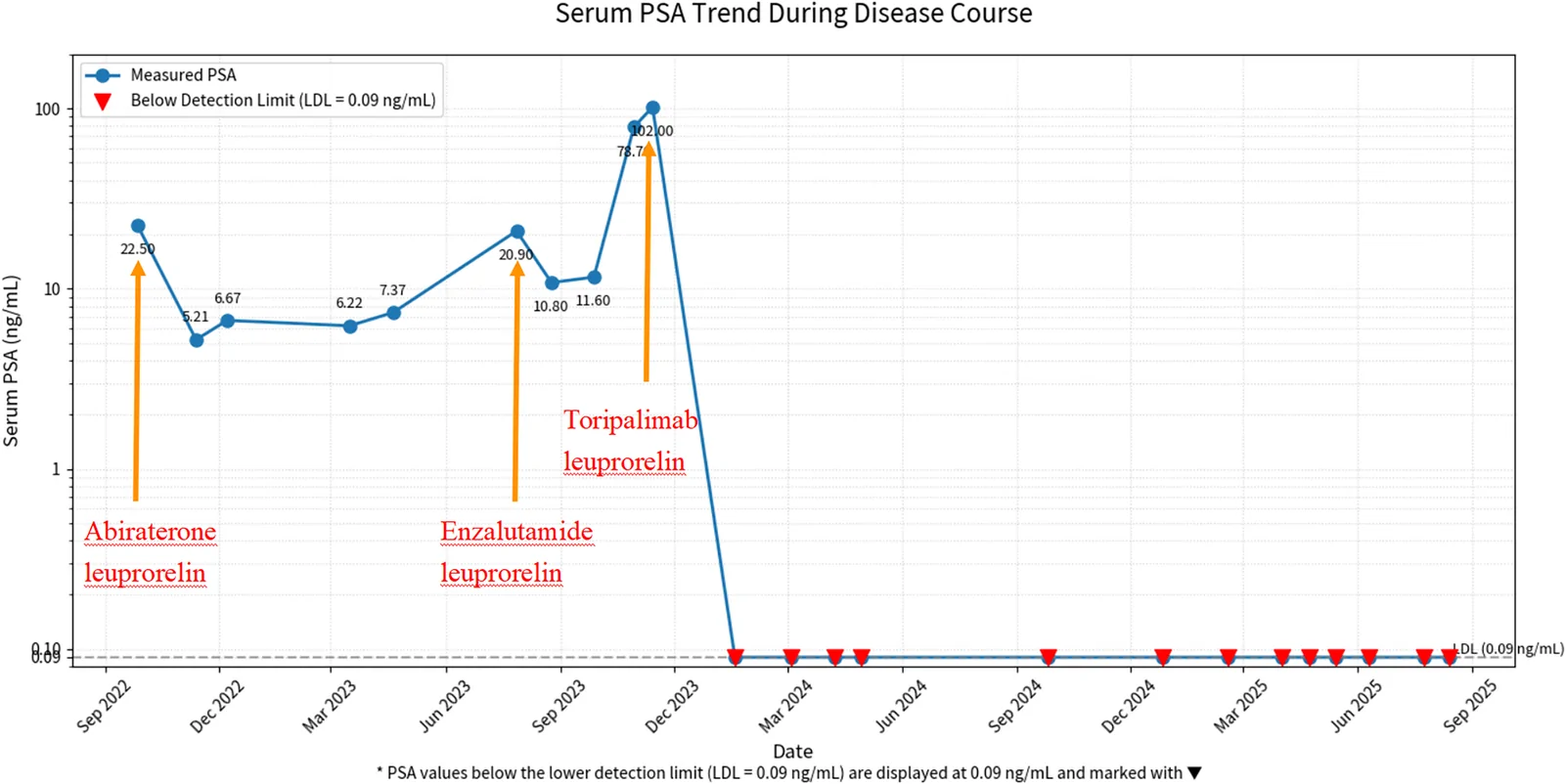
Serum PSA trend during the disease course.

We evaluated treatment response separately for soft-tissue, nodal, and bone compartments. RECIST version 1.1 criteria were applied only to measurable soft-tissue lesions; partial response was defined as ≥30% reduction in target-lesion diameter relative to baseline, with non-nodal lesions requiring a minimum long-axis diameter of 10 mm and pathological lymph nodes a minimum short-axis diameter of 15 mm.

Since serial MRI measurements were available for the locally invasive prostate-soft-tissue mass, we selected it as our sole non-nodal target lesion. Its maximal diameter decreased from 119 mm before toripalimab to 43 mm after six cycles and 31 mm after 25 cycles. Pelvic lymph nodes could not be selected as target lesions because radiology reports lacked consistent short-axis measurements; these nodes were classified as non-target disease and resolved on imaging after six cycles.

Bone lesions without soft-tissue components are non-measurable by RECIST 1.1 and were excluded from response assessment. Because bone scans were obtained as routine clinical studies rather than under a standardized PCWG-3 protocol, full PCWG-3 criteria could not be applied. We therefore interpreted bone scan results according to PCWG-3 principles, emphasizing newly emerging and confirmed lesions over isolated changes in radiotracer uptake.

The rib tracer-avid foci on the July 2023 bone scan were not verified by concurrent CT, MRI, SPECT/CT, or PSMA PET/CT; we therefore classify them as suspicious scan findings rather than definitive metastases. Bilateral iliac lesions identified on MRI, in contrast, represent confirmed bone metastases.

**Table 1** summarizes longitudinal PSA levels, therapeutic adjustments, quantitative MRI parameters, and bone-scan findings across the disease course. **Figure 4** summarizes the clinical timeline of treatment changes, PSA values, and imaging findings.

**Table 1:** Quantitative MRI measurements and imaging response across the disease course.

Time point/phase	PSA (ng/mL)	Quantitative MRI measurement	Pelvic lymph nodes	Bone imaging/response context
September 2022 Initial diagnosis	22.50	Reported prostate/soft-tissue dimensions: 63 × 59 × 81 mm.	Enlarged right pelvic sidewall nodes; short-axis measurements not reported.	Bone scintigraphy: no definite osseous metastasis.
July 2023 First progression	20.90	Reported dimensions: 45 × 71 × 64 mm; increased local invasion described.	Markedly enlarged pelvic nodes; short-axis measurements not reported.	Bone scan: new right 4th/8th and left 8th-9th rib foci suspicious for metastases; no contemporaneous confirmatory CT/MRI/SPECT-CT/PSMA PET-CT.
November 2023 Pre-toripalimab RECIST baseline	102	109 × 95 × 119 mm; maximum reported diameter 119 mm. This lesion was designated as the single non-nodal RECIST target lesion.	Enlarged pelvic metastatic nodes; short-axis measurements not reported, therefore assessed as non-target disease.	MRI: new bilateral iliac bone metastases. Bone disease excluded from RECIST measurement.
April 2024 After 6 cycles	<0.09	43 × 23 × 31 mm; maximum target-lesion diameter 43 mm (63.9% decrease from baseline).	Previously enlarged pelvic nodes no longer radiographically evident.	Bilateral iliac bone metastases radiologically stable.
June 2025 After 25 cycles	Undetectable	31 × 20 × 22 mm; maximum target-lesion diameter 31 mm (73.9% decrease from baseline).	Pelvic nodal non-target disease remained resolved.	Iliac bone lesions stable. Bone scan: no new metastatic lesions; prior suspicious rib uptake reduced/normalized.
After 31 cycles Latest outcome reported	<0.09	No radiographic progression reported; no additional lesion-specific measurement was available.	No nodal progression reported.	No clinical or radiographic progression reported. Toripalimab and leuprorelin remain ongoing at manuscript revision.

**Figure 4:**
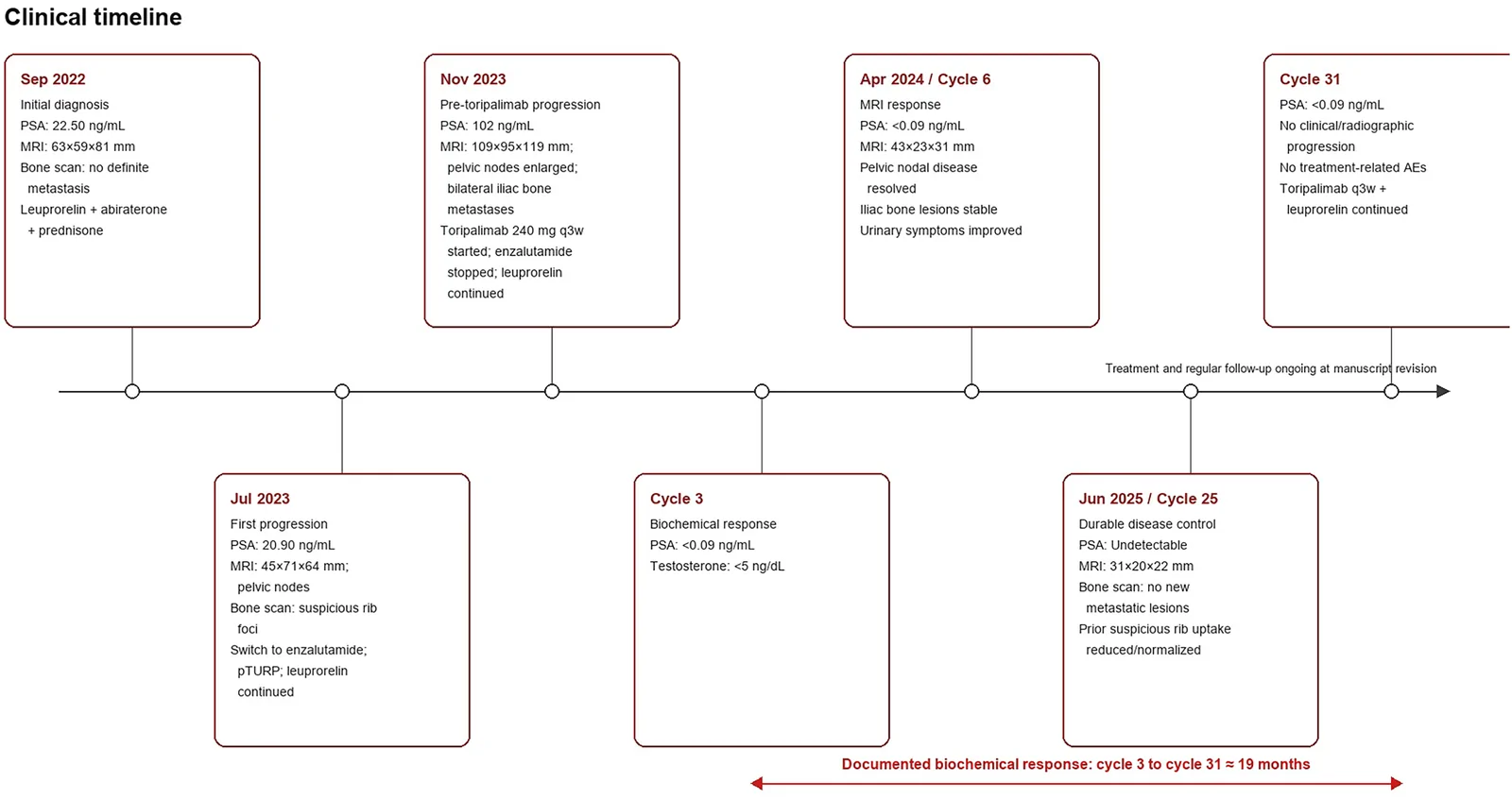
Clinical timeline summarizing PSA kinetics, MRI findings, bone-imaging findings, and systemic treatment changes: The timeline is not drawn to scale. The approximately 19-month response duration is calculated from the first documented biochemical response after cycle 3 through the cycle-31 assessment, based on regular 21-day toripalimab dosing without treatment delays.

## Patient perspective

After toripalimab therapy, the patient experienced marked relief of obstructive urinary symptoms and hematuria, and spontaneous voiding was restored after removal of the suprapubic catheter. The patient and his family considered the improvement in urinary function and disease control clinically meaningful. They were informed of the clinical significance and potential limitations of this case report and provided written consent for the publication of the relevant clinical information and accompanying images.

## Targeted narrative literature review

While pembrolizumab is included in EAU and NCCN recommendations for selected patients with prostate cancer on the basis of molecular biomarkers (6, 7), real-world clinical data for alternative PD-1/PD-L1 inhibitors in mCRPC remain sparse. We therefore performed a targeted narrative search of PubMed through September 2025 using combinations of the terms “prostate cancer”, “metastatic castration-resistant prostate cancer”, “mCRPC”, “PD-1”, “PD-L1”, “toripalimab”, “tislelizumab”, and “nivolumab”. We included case reports and case series describing individual patients with mCRPC treated with a non-pembrolizumab PD-1/PD-L1 inhibitor that provided patient-level treatment or outcome data. We excluded pembrolizumab-only reports, trials lacking individual-patient data, reviews, and publications without relevant clinical outcomes. No language restriction was applied. This review aimed to provide contextual background rather than a formal systematic review. We did not pre-register screening protocols or keep full screening logs, so we cannot report total screened or excluded records. Twelve published cases involving toripalimab, tislelizumab, or nivolumab were identified (**Table 2**) (5) (8–15). Three cases showed substantial PSA reductions after immunotherapy, whereas only one nivolumab-treated case reported a partial response according to RECIST version 1.1. Radiographic progression was also reported with each of the three agents. Immune-related adverse events occurred in some toripalimab- and nivolumab-treated patients, including grade 3–4 toxicities leading to treatment discontinuation. Importantly, several included papers focused primarily on immune-related adverse events instead of anti-tumor activity. Their clinical outcomes are only described narratively and cannot support cross-drug efficacy comparison.

**Table 2:** Review of prostate malignancy case reports and case series.

Study	Age	Family history of malignancy	Immunotherapy	Pathological type​	Mutation type	PSA level before immunotherapy (ng/ml)​​	Imaging based metastatic status prior to immunotherapy​	Immunotherapy interval (weeks)	Immunotherapy cycles	PSA response after immunotherapy (ng/ml)​	Adverse events	Tumor response evaluation after immunotherapy (per RECIST 1.1)​​​	Death
Ferreira Bruzzi Porto H, 2024 (8)	76	None​	Nivolumab	Neuroendocrine prostate carcinoma	TMB:9Muts/Mb, ATM, SF3B1 (G742D)	Undetectable PSA levels	Precaval chain and left inguinal, a left pelvic soft tissue mass, and several bone implants	3	4	Undetectable PSA levels	Harada like syndrome	Progressive disease	No
Zhao D, 2023 (9)	65	None​	Tislelizumab	Prostatic adenocarcinoma	TP53, BRCA1, BRCA2	105.5	Hepatic metastases​​	NA	NA	279.2	None​	Progressive disease	Yes
Liu SJ, 2022 (10)	68	NA	Toripalimab	Prostatic adenocarcinoma	NA	24.0	Extensive bone metastases (no imaging available)​	3	4	0.47	None​	NA	No
Liu SJ, 2022 (10)	79	NA	Toripalimab	Prostatic adenocarcinoma	NA	9.939	Extensive bone metastases (no imaging available)​	3	4	168.5	None​	NA	No
Liu SJ, 2022 (10)	69	NA	Toripalimab	Prostatic adenocarcinoma	NA	13.31	Extensive bone metastases (no imaging available)​	3	4	39.3	None​	NA	No
Liu SJ, 2022 (10)	82	NA	Toripalimab	Prostatic adenocarcinoma	NA	36.0	Extensive bone metastases (no imaging available)​	3	4	350.2	None​	NA	No
Kan S, 2021 (5)	78	NA	Toripalimab	Prostatic adenocarcinoma	NA	767	Extensive bone metastases (no imaging available)​	3	5	0.035	Lichenoid drug eruption on the lower lip	NA	No
Williams KJ, 2021 (11)	69	NA	Nivolumab	Prostatic adenocarcinoma	NA	NA	Multifocal metastatic disease (no imaging available)​	3	2	NA	Myositis and myasthenic syndrome.	NA	Yes
Henderson D, 2020 (12)	62	NA	Nivolumab	NA	NA	NA	Bone metastasis (unspecified)	NA	2	NA	Grade 3 diarrhoea and grade 2 immunotherapy-associated thyroiditis, large vessel vasculitis	Progressive disease	No
Zhang HC, 2019 (13)	79	Colon cancer of the patient’s father and brother.	Nivolumab	Prostatic adenocarcinoma	NA	NA	Pelvic and right femur metastases	9 → 3​	3	NA	ICPI-associated enterocolitis, abnormal liver biochemical results	NA	No
Monge C, 2018 (14)	79	NA	Nivolumab	NA	NA	NA	Bone metastasis (unspecified)	2	4	NA	Autoimmune myositis and myocarditis	NA	No
Basnet A, 2017 (15)	65	NA	Nivolumab	Prostatic adenocarcinoma	MSH2, MSH6	13.4	Progression of right pelvic bone metastasis​	NA	19	Undetectable PSA levels	None	Partial response	No

#NA not available, #9 → 3:The second cycle was administered 9 weeks after the first cycle, and the third cycle was administered 3 weeks thereafter.

## Discussion

Traditional management of mCRPC primarily relies on endocrine therapy, chemotherapy, and radionuclide therapy, etc. The advent of immune checkpoint inhibitors targeting the PD-1/PD-L1 pathway has revolutionized the treatment of various advanced cancers. However, although monotherapy with anti-PD-1/PD-L1 agents has been shown to improve the prognosis of some tumors, their clinical benefit remains limited in an unselected population of mCRPC patients.

A widely recognized explanation for the poor clinical activity of checkpoint inhibitors is that most patients with mCRPC have a tumor microenvironment with immunosuppression, which is characterized by low infiltration of CD8-positive T cells and increased presence of immunosuppressive cell types, including neutrophils, monocytic myeloid-derived suppressor cells, as well as TH17 and regulatory T (Treg) cell populations. Consequently, ongoing clinical trials are evaluating the potential of combining anti-PD-1/PD-L1 agents (e.g., pembrolizumab, toripalimab, nivolumab, durvalumab, avelumab, and atezolizumab) with established anticancer therapies (e.g., docetaxel and enzalutamide) to promote a more immunoreactive tumor microenvironment in prostate cancer. This includes combinations with inhibitors of other immune checkpoint pathways or additional systemic anticancer treatments (17).

The KEYNOTE-991 trial demonstrated that the addition of pembrolizumab to androgen deprivation therapy (ADT) combined with enzalutamide did not result in a statistically significant improvement in radiographic progression-free survival (rPFS) compared with ADT plus enzalutamide and placebo (18). The KEYNOTE-028 trial indicated that pembrolizumab did not show clinically meaningful benefit in heavily pretreated patients with mCRPC (19). KEYNOTE-199, KEYNOTE-365, and CheckMate 9KD reported that immune checkpoint inhibitor combinations may have activity in selected mCRPC settings, although results differ by partner therapy and biomarker context (20–22). A meta-analysis of 13 studies involving 2,553 patients with mCRPC suggested that PD-1/PD-L1 inhibitor-based regimens may improve progression-free survival, whereas an overall-survival benefit remains uncertain (17). Evidence for CTLA-4 blockade is likewise limited: a phase II study of ipilimumab in 10 patients with metastatic prostate cancer was terminated at interim analysis for failure to meet the primary endpoint, although 30% of patients experienced a PSA decline greater than 50% (16). Because ipilimumab targets CTLA-4 rather than PD-1/PD-L1, this study is discussed here as broader immunotherapy context rather than being included in the case-report review table.

The present case achieved durable biochemical and radiographic disease control without treatment-related adverse events. The most likely explanation is biomarker selection: the tumor was MSI-H and TMB-H (91.5 mutations/Mb), features associated with increased tumor immunogenicity and sensitivity to PD-1 blockade. Leuprorelin was continued throughout toripalimab treatment and maintained castrate testosterone levels, whereas enzalutamide was discontinued at toripalimab initiation. The patient had also undergone pTURP before immunotherapy; however, biochemical and radiographic progression to PSA 102 ng/mL with marked local, nodal, and osseous progression was documented after pTURP and immediately before toripalimab. This patient received continuous ADT and prior pTURP. For this single-case study, we cannot attribute tumor control solely to toripalimab. Good treatment compliance helps ensure adequate drug exposure, yet it cannot explain how strong or how long the anti-tumor effect lasted.

The primary limitation of this case report relates to the initial management of prostate cancer. The patient declined radical prostatectomy despite fulfilling surgical eligibility criteria and opted for medical therapy instead. Bone-modifying agents were not initiated when osseous metastases were subsequently identified. Other limitations deserve consideration. First, the suspicious rib lesions identified in July 2023 bone scan had no confirmatory cross-sectional or molecular imaging. Second, response assessment was done retrospectively using archived reports. Although we obtained sequential measurements for the prostate-soft-tissue mass, archived radiology reports did not record short-axis diameters for pelvic lymph nodes. For this reason, lymph nodes could not be selected as RECIST target lesions and were evaluated as non-target disease. Bone scans were ordered as routine clinical tests without standardized PCWG-3 scheduling, so we could not strictly apply PCWG-3 criteria. These limitations preclude definitive RECIST-based response classification and should be considered when interpreting the imaging findings.

In the reported treatment course, the patient had not received docetaxel or cabazitaxel before toripalimab and therefore should not be characterized as chemotherapy-refractory. For patients with mCRPC, particularly after progression on androgen-receptor pathway-directed therapy, comprehensive genomic profiling may identify biomarker-defined subgroups—such as MSI-H/dMMR or TMB-H disease—that are candidates for PD-1 blockade. The present case supports biomarker-guided consideration of immune checkpoint inhibition rather than broad use of toripalimab in unselected mCRPC. A thorough pretreatment and on-treatment evaluation remains essential to monitor immune-related and other treatment-associated adverse events.

## Conclusion

This biomarker-selected case supports the clinical value of comprehensive tumor genomic profiling in mCRPC. The durable biochemical and radiographic disease control observed during toripalimab treatment suggests that MSI-H/TMB-high prostate cancer is responsive to PD-1 blockade, including toripalimab. The patient remains on toripalimab and androgen-deprivation therapy with ongoing follow-up at the time of manuscript revision. Since this is only one patient who received continuous ADT and prior local surgery, these observations remain exploratory and should not be generalized to unselected mCRPC populations. We cannot extend these results to unselected mCRPC patients to claim general toripalimab efficacy.

## Data Availability

The original contributions presented in the study are included in the article/Supplementary Material. Further inquiries can be directed to the corresponding authors.
